# Dental environmental stressors and their association with perceived stress among undergraduate dental students at a Palestinian University: a cross-sectional study using DES and PSS-10

**DOI:** 10.3389/froh.2026.1952246

**Published:** 2026-09-28

**Authors:** Laith Alaqra, Thikrayat Badrasawi, Hasna Makkawi, Nabil Massad

**Affiliations:** 1 Faculty of Dentistry, An-Najah National University, Nablus, Palestine; 2 Public Health PhD Program, Faculty of Graduate Studies, Al-Quds University, Jerusalem, Palestine

**Keywords:** academic stress, dental environment stress scale, dental students (MESH), health profession education, Palestine, Perceived Stress Scale (PSS-10), student wellbeing

## Abstract

**Background:**

Dental education is associated with considerable academic, clinical, and psychological stress. This study aimed to assess perceived stress levels among undergraduate dental students in Palestine and identify dental environmental stressors associated with perceived stress.

**Methods:**

A cross-sectional study was conducted among undergraduate dental students in the first to fourth academic years at An-Najah National University. A total population sampling approach was used, and all eligible students were invited to participate. Data were collected using a self-administered electronic questionnaire that included demographic and health-related variables, the Perceived Stress Scale-10 (PSS-10), and the Arabic 41-item Dental Environment Stress (DES) scale. Descriptive statistics, Pearson's chi-square or Fisher's exact tests, non-parametric comparisons, Spearman's correlation, and multiple linear regression were performed.

**Results:**

Of 950 invited students, 635 valid responses were included in the final analysis. The mean PSS-10 score was 24.50 ± 5.88, with 61.4% of students reporting moderate stress and 35.4% reporting high stress. The DES total item mean was 1.53 ± 0.50, with 66.3% reporting moderate dental environmental stress. Internal consistency was good for PSS-10 (Cronbach's alpha = 0.833) and excellent for the modified DES scale (Cronbach's alpha = 0.928). The DES score was moderately correlated with the PSS-10 score (rho = 0.554, *p* < 0.001). In multiple linear regression, DES score showed the strongest independent association with PSS-10 score (B = 5.91, 95% CI: 5.09–6.72, *p* < 0.001). Female sex and year of study were also significantly associated with PSS-10 score.

**Conclusion:**

Perceived stress was common among dental students, with dental environmental stressors strongly associated with overall perceived stress. Workload, self-efficacy, female sex, and academic stage should be considered when designing student support and stress-reduction interventions.

## 1 Introduction

Stress is often characterized as the body's non-specific response to any strain placed on it and has major psychological, emotional, and physical consequences (1). Professionals in high-pressure occupations, such as dentistry, are frequently exposed to stressors that might influence their general well-being and professional performance (2). It is well documented that dental students often report higher levels of stress than students in other disciplines. Dentistry is often characterized as one of the most demanding specialties. This pattern continues after graduation (3). Dental students experience stress due to a number of variables, including a severe workload, the pressure to achieve high academic performance, the difficulties of scheduling and following up with patients, and personal and interpersonal problems (4). These stressors may arise from academic and non-academic sources and are called stresses (5)*.* The cumulative effect of these pressures has been documented to adversely affect the mental and physical health of students (6). Studies have shown that the amount of stress in the school environment is related to the academic performance, psychological and emotional well-being, physical health, and the formation of harmful habits like smoking and alcohol use (7).

To investigate stress levels in the dental educational environment, many tools can be utilized. The most common is the Perceived Stress Scale-10 (PSS-10), which is a 10-item survey that measures the degree to which situations in one's life are appraised as stressful. It has been validated as a reliable tool for measuring perceived stress in medical populations, with a reliability score exceeding 0.80 (8). Another commonly used tool in the dental educational environment is a modified version of the Dental Environment Stress (DES) survey. Originally developed by Garbee in 1981 (9), it consists of 41 items that capture stressors unique to predoctoral dental training. Evidence supporting the reliability of DES as a psychometric tool for measuring stress among dental students has been reported in previous studies. The DES and its modified or translated versions have been used in several countries, including Japan (10), Jordan (6), Saudi Arabia (4, 11), Brazil through the Portuguese/Lusophone version (5), and Croatia (4). Evidence from systematic reviews also indicates that dental students have at least moderate levels of perceived stress, and some studies report even higher levels of perceived stress (7). However, generalization of these findings across populations is complicated by individual susceptibility to stress and social, cultural, and demographic factors (12). For example, gender has been shown consistently to influence the perception of stress, with females generally reporting higher levels of stress than males (1, 2, 6, 11).

These differences are particularly evident among Palestinians living under occupation. Political instability, alongside social and economic restrictions, directly or indirectly shapes the dental education environment and affects students' stress levels (13). Evidence from conflict-affected settings has shown increased psychological distress and mental health problems among populations exposed to prolonged instability and war conditions (14), which can reasonably be expected to have a similar impact in Palestine. In addition, studies from countries affected by conflict, including Yemen and Syria, indicate that social, economic, and political pressures can negatively affect dental education and the preparation of students for oral healthcare practice (13, 15).‏ Although previous international studies have combined DES with general perceived stress measures, evidence from Palestine remains limited. This study aimed to assess perceived stress levels among dental students in Palestine, identify key stress-inducing factors within their educational environments, and examine how these stressors were associated with perceived stress. The findings provide insight into stress patterns among Palestinian dental students and may inform strategies to support their well-being in Palestine.

## 2 Materials and methods

### 2.1 Study design and population

A cross-sectional design was used to assess stress levels and potential stressors in the dental education environment at An-Najah National University, Palestine. The study targeted all eligible undergraduate dental students enrolled in the first, second, third, and fourth years. Data were collected between April 2025 and May 2025. The target population of 950 students represented all undergraduate dental students enrolled in the Faculty of Dentistry during the study period. Because the faculty was newly established, no fifth-year cohort was enrolled at that time; therefore, the first to fourth academic years represented the full eligible undergraduate dental student population. A total population sampling approach was used, whereby all students in the target population were invited to participate. Students were excluded if they were outside the target academic programme, declined consent, or submitted incomplete questionnaires. A questionnaire was considered valid if the participant met the eligibility criteria, provided electronic informed consent, and provided the essential variables and scale data required to calculate the PSS-10 total score and the DES total item mean score. Data were collected using a structured self-administered questionnaire distributed electronically through university emails and WhatsApp groups according to students' preferred communication channels. Duplicate electronic submissions were minimized by restricting the questionnaire to one response per institutional email account and by screening responses for duplicates before analysis.

### 2.2 Measurement tool and data collection

Data were collected through a self-administered survey distributed to participants who provided consent. The survey consisted of three sections. The first covered demographic and health-related information, including sex, age, residence, marital status, sleeping hours, smoking habits, physical activity, and self-reported medical conditions. Medical conditions were assessed using a yes/no item asking whether the participant had any diagnosed health condition. Body mass index (BMI) was calculated from self-reported weight and height as weight in kilograms divided by height in meters squared (kg/m^2^). BMI was categorized as underweight (<18.5 kg/m^2^), healthy weight (18.5–24.9 kg/m^2^), overweight (25.0–29.9 kg/m^2^), and obesity (≥30.0 kg/m^2^). The second and third sections included the DES scale and the PSS-10, respectively.

#### 2.2.1 Dental environment stress (Des) scale

The Arabic 41-item DES scale used in this study to assess stressors specific to the dental educational environment was adapted from the 41-item modified DES used by Al-Sowygh et al. (16), without adding, deleting, or modifying items, who modified the original DES for use among Saudi dental students, retaining 25 items from the original DES and 16 additional items based on review of modified DES versions previously published in the literature (2, 5, 6, 16–18). The questionnaire was translated into Arabic, and its reliability and validity were assessed by the original authors. The seven-domain structure used in the present study followed the domains reported by Al-Sowygh et al.: “Self-efficacy,” “Faculty and administration,” “Workload,” “Patient Treatment,” “Clinical Training,” “Performance pressure,” and “Social Stressors” (16). The responses to each item were rated on a four-point scale (0 = not stressful, 1 = mildly stressful, 2 = moderately stressful, 3 = very stressful) and a fifth option of not applicable. Responses marked ‘Not applicable’ were recoded as missing values and excluded from subscale mean calculations to avoid diluting the stress scores. Conceptually, “Not applicable” does not represent a level of stress but rather indicates that the student was not exposed to that specific stressor, not absence of stress; coding them as zero would have artificially diluted the DES score. Therefore, treating it as missing ensured that domain scores reflected stress levels among students who experienced the relevant stressors. For descriptive analysis, the DES total item mean score was categorized according to the DES response-scale anchors as low stress = 0–1, moderate stress =  > 1–2, and high stress =  > 2–3. These categories were operationally derived from the four-point DES scoring scale and were used to facilitate descriptive interpretation and cross-tabulation; they were not intended to represent externally validated diagnostic thresholds. In the current study, the internal consistency of the modified 41-item DES was assessed using Cronbach's alpha.

#### 2.2.2 Perceived stress scale-10 (Pss-10)

The PSS-10 is a widely used self-report tool assessing the degree to which individuals perceive situations in their lives as stressful. The PSS-10 was developed by Cohen et al. in 1983 and exhibited satisfactory reliability (19). It measures how individuals view their lives as unpredictable, unmanageable, and burdened by excessive obligations. Each item was rated on a 5-point Likert scale, ranging from 0 (never) to 4 (very often). The total PSS-10 score was calculated by summing the scores of all ten items after reverse-scoring the positively worded items (items 4, 5, 7, and 8). Based on the standard interpretation of the PSS-10 total score, the overall score ranged from 0 to 40, with higher scores indicating greater perceived stress levels. Scores were categorized as 0-13 for low stress, 14-26 for moderate stress, and 27-40 for high stress (20). An Arabic version of PSS that has demonstrated acceptable reliability and validity was used (21, 22).

### 2.3 Data analysis

Data were analyzed using IBM SPSS Statistics version 26. Descriptive statistics were performed to summarize participants' characteristics and study variables. Categorical variables were presented as frequencies and percentages, while continuous variables were summarized using appropriate measures of central tendency and dispersion. Normality was assessed before selecting the appropriate statistical tests (by histograms, Q-Q plots, skewness and kurtosis values, and normality tests). Internal consistency of the PSS-10 and modified DES scales was evaluated using Cronbach's alpha. For the modified DES, “Not applicable” responses were treated as missing rather than zero, and Cronbach's alpha was calculated using pairwise available responses, meaning that each item-pair correlation was based on participants who had valid responses for both items. The PSS-10 score was analyzed both as a continuous score and as categorized stress levels. Associations between PSS-10 stress categories and sociodemographic, lifestyle, academic, and DES variables were examined using the chi-square test. When chi-square assumptions were not met due to small expected cell counts, Fisher's exact test was used. For comparisons of continuous PSS-10 scores across participant groups, non-parametric tests were used where appropriate, including the Mann–Whitney U test for two-group comparisons and the Kruskal–Wallis test for variables with more than two groups. Spearman's correlation was used to assess the relationship between continuous PSS-10 scores and DES total and domain scores. Multiple linear regression was performed to examine factors associated with continuous PSS-10 scores. Variables were considered for inclusion in the regression model based on theoretical relevance and bivariate associations with PSS at *p* < 0.10. Age was not included because of its conceptual overlap with year of study and the very small number of participants aged >25 years. The year of study was retained instead because it was more directly relevant to the academic stage in dental education. Regression assumptions were assessed by checking multicollinearity, residual normality, and homoscedasticity, and results were reported as coefficients with 95% confidence intervals. A p-value of <0.05 was considered statistically significant.

### 2.4 Ethical approval

This study was conducted in accordance with the ethical principles of the Declaration of Helsinki. Ethical approval was obtained from the Institutional Review Board at An-Najah National University on July 2024, reference number [Dent. Med July. 2024/15]. For the electronic questionnaire, the first page included an information sheet explaining the study purpose, voluntary participation, confidentiality, and the right to withdraw. All participants provided electronic informed consent by selecting the consent option before proceeding to the questionnaire. The collected information was anonymized and coded securely with limited access to the main investigator and reported in aggregate form only.

## 3 Results

A total of 950 students were invited to participate, of whom 636 responded, giving an overall response rate of 66.9%. After excluding one response with incomplete DES data, 635 valid responses were included in the final analysis. The detailed distribution of participants' demographic and health characteristics is presented in Table 1.

**Table 1:** Demographic and general health characteristics.

Variable	Category	n	%
Age category	<20 years	249	39.2
	20–25 years	383	60.3
	>25 years	3	0.5
Sex	Female	506	79.7
	Male	129	20.3
Place of residency	Northern governorates	555	87.4
	Central governorates	20	3.1
	Southern governorates	15	2.4
	Palestinian interior	45	7.1
Marital status	Single	628	98.9
	Married	7	1.1
BMI category	Underweight	65	10.2
	Healthy weight	413	65.0
	Overweight	126	19.8
	Obesity	31	4.9
Year of study	First year	158	24.9
	Second year	214	33.7
	Third year	134	21.1
	Fourth year	129	20.3
Sleep hours	<6 hours	194	30.6
	6–8 hours	395	62.2
	>8 hours	46	7.2
Smoking	No	535	84.3
	Yes	100	15.7
Sport practice	No	495	78.0
	Yes	140	22.0
Medical conditions	No	602	94.8
	Yes	33	5.2

Internal consistency was good for the PSS-10 (Cronbach's alpha = 0.833) and excellent for the modified 41-item DES (Cronbach's alpha = 0.928 using pairwise available responses, with “Not applicable” treated as missing). These results indicate that both scales were reliable for assessing perceived stress and dental environmental stress in the current sample. Histograms and Q-Q plots were used to test the normality assumption and showed that the data were mostly evenly spread out. However, non-parametric tests were used for bivariate analyses when they were needed. Analysis of PSS and DES responses revealed that almost two-thirds of students experienced moderate levels of stress on both scales. (Table 2, Figure 1).

**Table 2:** Summary of PSS-10 and DES scores and stress level distribution.

Measure	PSS-10 perceived stress	DES total item means
Valid n	635	635
Mean ± SD	24.50 ± 5.88	1.53 ± 0.50
Median (IQR)	25 (21–28)	1.53 (1.19–1.87)
Range	6–40	0.14–3.00
Stress level distribution		
Low stress	20 (3.2%)	102 (16.1%)
Moderate stress	390 (61.4%)	421 (66.3%)
High stress	225 (35.4%)	112 (17.6%)

**Figure 1:**
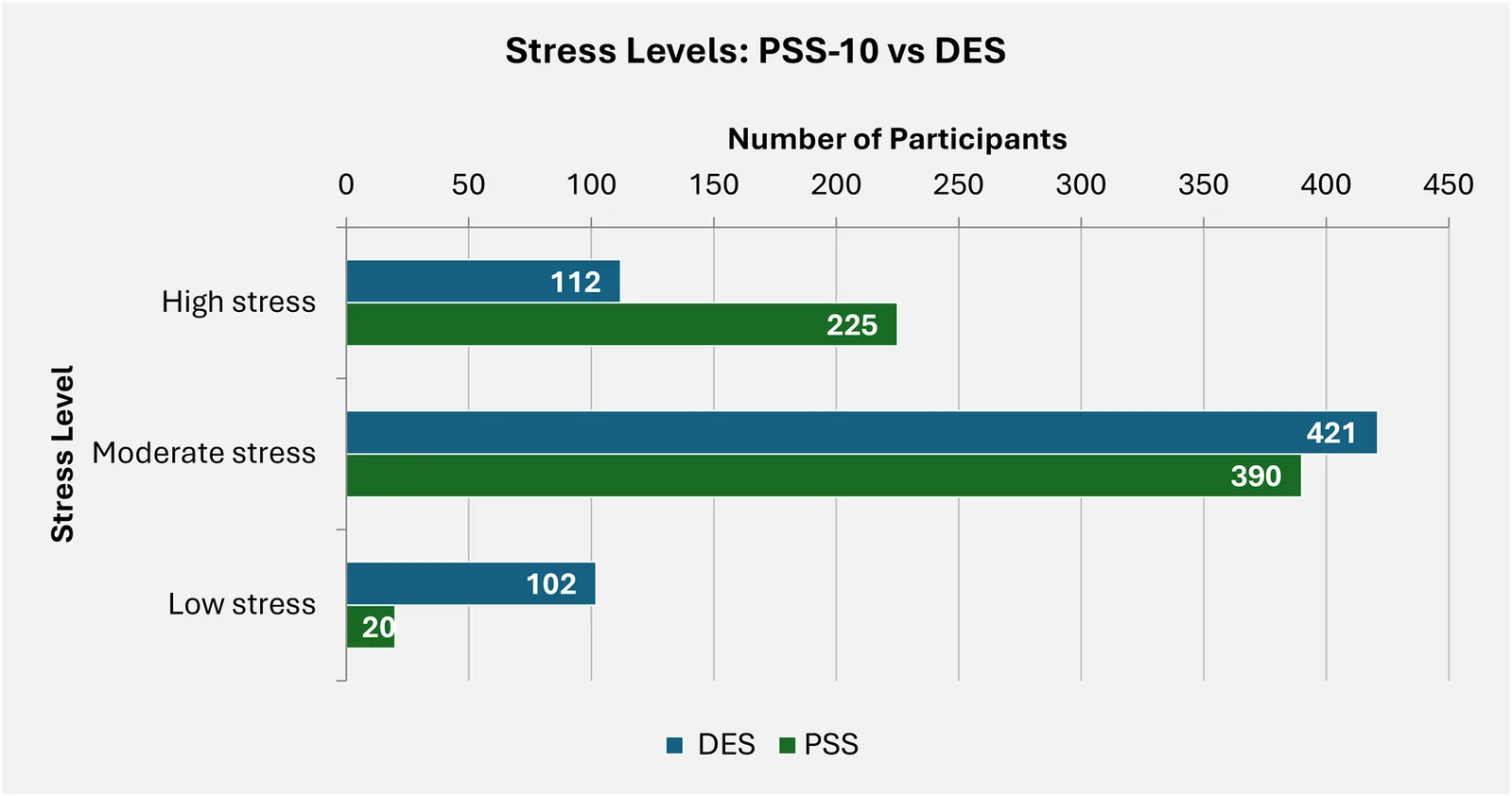
Distribution of stress levels according to PSS-10 and DES scoring.

The association between PSS-10 stress categories and sociodemographic, lifestyle, academic, and DES variables was assessed using Pearson's chi-square test or Fisher's exact when expected cell counts were small. PSS-10 scores were categorized into low, moderate, and high perceived stress levels, and cross-tabulations were performed to examine whether the distribution of stress levels differed across participant characteristics. Age, sex, year of study, physical activity, and DES stress category (*p* < .001) were significantly associated with PSS-10 stress categories, as shown in Table 3.

**Table 3:** Association of PSS-10 categories with sociodemographic, lifestyle, academic, and DES variables among dental students (*n* = 635).

Variable	Category	N (%)	PSS-10			P-value
			low stress n (%)	moderate stress n (%)	high stress n (%)	
Age	<20	249 (39.2)	6 (30.0)	137 (35.1)	106 (47.1)	0.021*
	20-25	383 (60.3)	14 (70.0)	250 (64.1)	119 (52.9)	
	>25	3 (0.5)	0 (0.0)	3 (0.8)	0 (0.0)	
Sex	Male	129 (20.3)	13 (65.0)	91 (23.3)	25 (11.1)	<0.001*
	Female	506 (79.7)	7 (35.0)	299 (76.7)	200 (88.9)	
Place of residency	Northern West Bank	555 (87.4)	16 (80.0)	340 (87.2)	199 (88.4)	0.491
	Middle West Bank	20 (3.1)	1 (5.0)	13 (3.3)	6 (2.7)	
	Southern West Bank	15 (2.4)	1 (5.0)	7 (1.8)	7 (3.1)	
	Palestinian interior	45 (7.1)	2 (10.0)	30 (7.7)	13 (5.8)	
Marital status	Single	628 (98.9)	20 (100.0)	386 (99.0)	222 (98.7)	0.769
	Married	7 (1.1)	0 (0.0)	4 (1.0)	3 (1.3)	
BMI	Underweight	65 (10.2)	1 (5.0)	44 (11.3)	20 (8.9)	0.291
	Healthy weight	413 (65.0)	10 (50.0)	255 (65.4)	148 (65.8)	
	Overweight	126 (19.8)	8 (40.0)	75 (19.2)	43 (19.1)	
	Obesity	31 (4.9)	1 (5.0)	16 (4.1)	14 (6.2)	
Sleeping hours	<6 hours	194 (30.6)	4 (20.0)	114 (29.2)	76 (33.8)	0.218
	6-8 hours	395 (62.2)	16 (80.0)	250 (64.1)	129 (57.3)	
	>8 hours	46 (7.2)	0 (0.0)	26 (6.7)	20 (8.9)	
Smoking	No	535 (84.3)	15 (75.0)	332 (85.1)	188 (83.6)	0.386
	Yes	100 (15.7)	5 (25.0)	58 (14.9)	37 (16.4)	
Physical activity	No	495 (78.0)	11 (55.0)	302 (77.4)	182 (80.9)	0.036*
	Yes	140 (22.0)	9 (45.0)	88 (22.6)	43 (19.1)	
Medical conditions	No	602 (94.8)	20 (100.0)	371 (95.1)	211 (93.8)	0.571
	Yes	33 (5.2)	0 (0.0)	19 (4.9)	14 (6.2)	
Year of study	First year	158 (24.9)	3 (15.0)	83 (21.3)	72 (32.0)	0.012*
	Second year	214 (33.7)	8 (40.0)	145 (37.2)	61 (27.1)	
	Third year	134 (21.1)	3 (15.0)	77 (19.7)	54 (24.0)	
	Fourth year	129 (20.3)	6 (30.0)	85 (21.8)	38 (16.9)	
DES	Low stress	102 (16.1)	9 (45.0)	86 (22.1)	7 (3.1)	<0.001*
	Moderate stress	421 (66.3)	11 (55.0)	266 (68.2)	144 (64.0)	
	High stress	112 (17.6)	0 (0.0)	38 (9.7)	74 (32.9)	

Values are presented as n(%). Percentages in PSS-10 columns are column percentages within each PSS stress category. PSS categories were defined as low (0–13), moderate (14–26), and high (27–40). DES categories were operationally derived and based on item mean cut-offs: 0–1 low, >1–2 moderate, and >2–3 high. *p < 0.05. The chi-square test was used when assumptions were met. Fisher's exact test was used when expected cell counts were small.

Further analysis using the Kruskal–Wallis test, followed by Dunn's post hoc pairwise comparisons with Bonferroni corrections, showed that Year I students had significantly higher PSS scores than Year II (*p* < .001) and Year IV (*p* = .021) students. Year III did not differ significantly from any other year despite having the second-highest mean score. (Supplementary Table 1). Pairwise chi-square comparisons with Bonferroni correction showed that all DES stress categories differed significantly in their distribution of PSS-10 stress categories (Supplementary Table 2).

Detailed findings related to the DES domains and the highest-ranked individual stressors are presented in Supplementary Table 3. These data provide additional information on the main sources of dental environmental stress among the participating students. The highest mean stress scores were observed in the workload domain, followed by performance pressure, indicating that academic burden, examinations, time limitations, and fear of failure were the most prominent sources of dental environmental stress among students. Patient treatment and self-efficacy-related stressors also contributed to the overall stress experience, but to a lesser extent.

Subsequently, Spearman's correlation showed a significant moderate positive correlation between continuous PSS-10 scores and continuous DES total item mean scores (rho = 0.554, *p* < 0.001), indicating that students with higher perceived stress also reported higher dental environmental stress levels. All seven DES domains were also significantly positively correlated with PSS-10 scores, with the strongest correlations observed for workload (rho = 0.547, *p* < 0.001) and self-efficacy (rho = 0.513, *p* < 0.001). (Figure 2).

**Figure 2:**
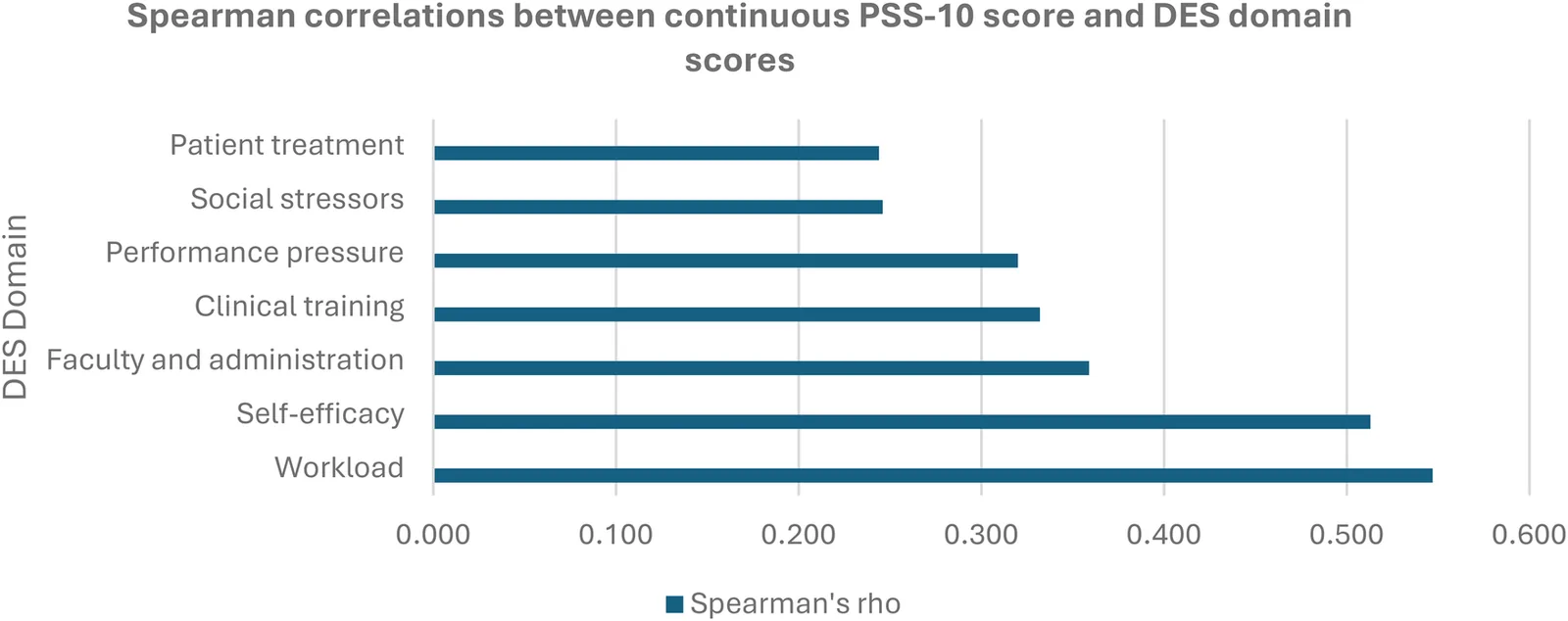
Spearman correlation between PSS-10 score and DES domains.

Multiple linear regression was performed to examine factors associated with continuous PSS-10 scores. The model was statistically significant and explained approximately one-third of the variance in perceived stress scores (R^2^ = 0.344, adjusted R^2^ = 0.336, *p* < 0.001). The DES total score showed the strongest independent association with the PSS-10 score. For each one-point increase in DES score, the PSS-10 score was higher by 5.91 points after adjustment for sex, year of study, sleep duration, and physical activity (95% CI: 5.09–6.72, *p* < 0.001). Female students also had significantly higher PSS-10 scores than male students by 2.37 points (95% CI: 1.28–3.46, *p* < 0.001). Compared with first-year students, second-year and fourth-year students had significantly lower PSS-10 scores, while sleep duration and physical activity were not significantly associated with the PSS-10 score after adjustment. Regression diagnostics showed no evidence of multicollinearity, with VIF values ranging from 1.04 to 1.60 and tolerance values ranging from 0.624 to 0.960. Residual normality was acceptable using the Shapiro–Wilk test (*p* = 0.128), and homoscedasticity was supported by a non-significant Breusch-Pagan test (*p* = 0.653).

## 4 Discussion

This study was the first of its kind to be conducted at a Palestinian university aiming to assess perceived stress levels among undergraduate dental students and to identify dental environmental and student-related factors associated with perceived stress. The findings showed that 96.9% of students reported moderate or high levels of perceived stress (61.4% and 35.4%, respectively). These findings are consistent with the findings of previous studies suggesting that dental students are usually exposed to high levels of stress during their study (1, 6, 18). The demanding nature of dentistry stems from factors such as the extensive syllabus, frequent examinations and quizzes, the challenge of learning and performing clinical or preclinical procedures, and the difficulty or anxiety associated with managing patients (23). The DES score showed the strongest independent association with PSS-10 score, indicating that stressors within the dental educational environment were closely linked to students' overall perceived stress. Female sex was also independently associated with higher PSS-10 scores, while second- and fourth-year students reported significantly lower perceived stress compared with first-year students. These findings support the view that stress among dental students is multifactorial and influenced by academic workload, educational environment, self-efficacy, gender-related factors, and stage of training.

Overall, the present findings are consistent with previous regional and international research showing that dental students experience considerable stress and that stress is strongly influenced by the demands of the dental educational environment (2, 24, 25). The strong association between DES total score and PSS-10 score in the current study highlights the important role of the dental educational environment in shaping students' perceived stress. This finding is consistent with the original work of Garbee et al., who reported that academic demands and faculty-student relationships were major sources of stress among dental students (9). It is also supported by the systematic review by Elani et al., which concluded that dental students experience considerable stress during training, mainly because of the demanding nature of dental education (7). The difference between PSS-10 and DES stress categories is expected because the two scales measure related but distinct constructs. PSS-10 assesses general perceived stress, while DES focuses on dental educational environment-specific stressors. Therefore, students may report low dental environmental stress but still experience moderate or high general stress due to personal, social, academic, or contextual factors (Figure 1). Thus, PSS-10 and DES provide complementary rather than identical information. However, because both DES and PSS-10 assess stress-related constructs and were measured simultaneously using self-reported scales, the strong association between them may partly reflect conceptual overlap. Therefore, the regression results should be interpreted as associations rather than evidence that dental environmental stressors predict or cause general perceived stress.

In this study, all DES domains were positively correlated with PSS-10 scores (Figure 2), but the most strongly correlated with perceived stress were workload and self-efficacy, suggesting that academic burden and students' confidence in managing educational and clinical requirements are central contributors to perceived stress. This finding is consistent with previous studies reporting workload and self-efficacy-related concerns as major stressors among dental students (2). In Saudi Arabia, Al-Sowygh et al. found relatively high perceived stress among dental students at King Saud University and identified female sex, advanced study level, and academic distress as important stress-related (2). Similarly, a recent Jordanian study among clinical dental students found that stress was influenced by academic performance, gender, year level, and the type and time required for clinical treatment, with completion of course requirements among the most commonly reported stressors (26). These findings are relevant to dental education, where students are required to integrate theoretical knowledge, manual skills, clinical decision-making, patient management, and time-bound academic assessments.

Female students had significantly higher perceived stress scores than male students, although some studies have shown no difference between male and female students (27). Our finding is consistent with several studies reporting higher stress among female dental students in both regional and international settings (13, 25, 28, 29). Halboub et al. found that female undergraduate dental students reported higher stress levels than males (13, 28). Hayes et al. also reported higher stress levels among female dental students in Canada (29). Similar gender differences were reported in studies using PSS-10 and DES-related tools among dental students (30, 31). The gender gap observed in these studies may be explained in terms of socio-cognitive behaviors. This, however, should be interpreted carefully, as females tend to be more expressive when discussing their stress and emotions, whereas males often display the opposite behaviour (32). Higher reported stress among female students may reflect differences in stress perception, willingness to report psychological distress, coping strategies, academic expectations, or gender-specific social and educational pressures, rather than an inherent biological vulnerability. This highlights the importance of providing a safe and supportive environment and gender-sensitive student counselling and mentoring systems while avoiding stigmatizing or overgeneralizing female students as a vulnerable group.

Previous studies showed that the level of perceived stress increased in relation to academic year, either peaking at the final year or at the transition from preclinical to clinical training (7, 33). Our study's bivariate and multivariate analyzes showed that first-year students had the highest perceived stress scores, significantly higher than both Year II (*p* = 0.006) and Year IV (*p* = 0.002). This suggests that the higher stress levels seen in first-year students may be due to other factors, most likely DES stress level and sex, which were found to be independently associated with PSS-10 score in the regression model, rather than being an effect of the study year itself. The heightened stress levels observed in first-year students may nonetheless be specifically attributed to the transition from school to university, separation from family, transportation difficulties, and the broader socio-political context in Palestine at the time of data collection. However, because this study did not directly measure conflict exposure, war-related stress, transportation barriers, or living arrangements, this explanation should be viewed as a possible contextual interpretation rather than evidence directly supported by the measured study variables. The lower adjusted PSS-10 scores among second- and fourth-year students compared with first-year students may reflect adaptation over time. Students who progress through the program may develop better familiarity with academic expectations, improved coping strategies, better adaptability with logistical challenges, stronger peer networks, and more confidence in managing dental school requirements. This interpretation is supported by literature emphasizing the role of coping and self-efficacy in dental student stress (2, 11, 18, 30, 33). Therefore, these results support the need for early preventive interventions such as orientation programs, peer mentoring, academic advising, and early psychological support, which may help students adapt before stress becomes chronic.

Although the significant bivariate association between physical activity and PSS-10 categories was consistent with previous studies (34), it wasn't independently associated with the PSS-10 score in the adjusted regression model. This suggests that the apparent association between physical activity and perceived stress may be partly explained by other factors, particularly DES score, sex, and year of study. In contrast to previous research that showed that students who had poor sleep quality experienced significantly higher levels of stress (35), in this study it was not significantly associated with PSS-10 score after adjustment. These findings do not mean that sleep and physical activity are unimportant for student well-being; rather, they indicate that, in this dataset, dental environmental stress had a stronger relationship with the PSS-10 score.

The findings have important implications for dental education. The DES score's strong independent association with perceived stress suggests that stress reduction should extend beyond individual-level advice. Instead, institutional changes are needed to address modifiable educational stressors. These may include reviewing workload distribution, coordinating examination schedules, ensuring transparent clinical requirements, providing timely feedback, strengthening student-faculty communication, and enhancing preclinical preparation before patient care. Stress-management interventions may also be beneficial, although evidence specific to dental education remains limited (36). A systematic review by Alzahem et al. found that relatively few studies have evaluated stress-management programs in dental students, but some interventions showed significant stress reduction and were perceived as useful by students (37). An additional contribution of this study is that it provides further evidence supporting the reliability and usefulness of the modified Arabic 41-item DES scale as a tool for assessing stressors within the dental educational environment. Similar translated or modified versions of the DES have shown acceptable psychometric properties in previous studies (4, 5). In the present study, the modified Arabic DES demonstrated excellent internal consistency, and its total score showed a significant positive association with PSS-10 scores. Although the PSS-10 measures general perceived stress and the DES assesses dental environment-specific stressors, the distribution of stress levels on both scales showed that most students experienced moderate stress on both scales (Figure 1).

Because of the cross-sectional design, the findings represent associations rather than cause-effect relationships. The study was conducted in one university, which may limit generalizability to other dental schools in Palestine or the region. In addition, non-response bias cannot be excluded because non-responders may have differed from responders in their perceived stress levels. Data were self-reported, which may introduce response bias. Although the Arabic DES demonstrated excellent internal consistency in the present sample, full psychometric validation was not performed. Future studies should further evaluate the construct validity and stability of the modified Arabic DES among Palestinian and regional dental student populations. Finally, unmeasured factors such as coping strategies, financial stress, social support, academic performance, and mental health history may have influenced perceived stress. In addition, treating “Not applicable” responses as missing may have resulted in unequal valid response counts across DES items and domains, which could affect the comparison of domain scores. On the other hand, this study targeted the full undergraduate dental student population within the faculty with a satisfactory sample size of 635 participants. It also assessed both general perceived stress and dental environment-specific stressors using established stress measures. Treating “Not applicable” DES responses as missing was also methodologically appropriate, because these responses indicate lack of exposure to a stressor rather than a low level of stress.

## 5 Conclusion

This study concluded that dental environmental stress, especially workload and self-efficacy-related stressors, strongly correlates with perceived stress among dental students. Female students reported higher perceived stress, and first-year students appeared to be particularly vulnerable compared with some senior groups. Effective stress-management programs, curricular review, and redesign, alongside longitudinal, multi-institutional studies, should be undertaken to better understand and minimize stress within the dental education environment.

## Data Availability

The original contributions presented in the study are included in the article/Supplementary Material, further inquiries can be directed to the corresponding authors.

## References

[1] Alamoush RA Al-Sawaeir S Baker DA Aljamani SA Alomoush SA MK A-O. Stress experienced by dental students performing clinical training in different dental disciplines: a cross-sectional study. J Occup Health. (2024) 66(1):uiae006. 10.1093/joccuh/uiae006 38348494 PMC10899752

[2] ZH A-S. Academic distress, perceived stress and coping strategies among dental students in Saudi arabia. Saudi Dent J. (2013) 25(3):97–105. 10.1016/j.sdentj.2013.05.002 24179318 PMC3809483

[3] Jena M Satyarup D Nagarajappa R Dhar U. Stress in dentistry: a review. Indian J Forensic Med Toxicol. (2020) 14(4):8667–70. 10.37506/ijfmt.v14i4.13066

[4] Laktić M Kuftinec K Čelebić A Kovačić I Nasser Alhajj M Peršić Kiršić S. Psychometric properties of the Croatian language version of the dental environment stress questionnaire on dental medicine students. Acta Stomatol Croat. (2017) 51(3):188–94. 10.15644/asc51/3/2 PMC5708330 29225359

[5] Sangiorgio JPM Araujo PM Navarro CH Zen IR Costa SC Ribeiro PHV Ferelle A Dezan-Garbelini CC, Dental environment stress: findings among lusophone dental students. Pesqui Bras Odontopediatria Clin Integr. (2016) 16(1):411–24. 10.4034/PBOCI.2016.161.43

[6] Alhajj MN Khader Y Murad AH Celebic A Halboub E Márquez JR Macizo CC Khan S Basnet BB Makzoumé JE De Sousa-Neto MD Camargo R Prasad DA Faheemuddin M Mir S Elkholy S Abdullah AG Ibrahim AA Al-Anesi MS Al-Basmi AA, Perceived sources of stress amongst dental students: a multicountry study. Eur J Dent Educ. (2018) 22(4):258–71. 10.1111/eje.12350 29607584

[7] Elani HW Allison PJ Kumar RA Mancini L Lambrou A Bedos C. A systematic review of stress in dental students. J Dent Educ. (2014) 78(2):226–42. 10.1002/j.0022-0337.2014.78.2.tb05673.x 24489030

[8] Shi C Guo Y Ma H Zhang M. Psychometric validation of the 14-item perceived stress scale in Chinese medical residents. Curr Psychol. (2019) 38(6):1428–34. 10.1007/s12144-019-00285-y

[9] Garbee WH Zucker SB Selby GR. Perceived sources of stress among dental students. J Am Dent Assoc. (1980) 100(6):853–7. 10.14219/jada.archive.1980.0279 6929835

[10] Liao SR Seki N Akiyama M Shinada K Morio I. Perceived stress and career planning awareness of Japanese and Taiwanese undergraduate dental hygiene students. J Dent Sci. (2023) 18(3):1094–102. 10.1016/j.jds.2022.11.012 37404668 PMC10316415

[11] Khanagar SB Al-Ehaideb A Jamleh A Ababneh K Maganur PC Vishwanathaiah S Awawdeh MA Naik S Al-Kheraif AA Bhandi S Zanza A Testarelli L Patil S, Psychological distress among undergraduate dental students in Saudi arabia and its coping strategies: a systematic review. Healthcare. (2021) 9(4):429. 10.3390/healthcare9040429 33917191 PMC8068084

[12] Arheiam AA Tantawi ME Al-Omami M Peeran SW Elmisalati W. Perceptions of stress among dental students living in a war-affected zone. J Dent Educ. (2019) 83(1):48–55. 10.21815/JDE.019.006 30600249

[13] Halboub E Alhajj MN Al Wesabi MA Al Sanaani S Mufadhal A. Dental environment and war-related stress among dental students, Yemen. East Mediterr Heal J. (2019) 25(8):529–36. 10.26719/emhj.19.002 31612966

[14] World Health Organization. Annual Report 2015: Syrian Arab Republic. Cairo: World Health Organization, Regional Office for the Eastern Mediterranean (2015).

[15] Shehada MR Alfakhry G Jamous I Aljoujou AA Abdulhak M. Major stress sources amongst dental students at Damascus university, Syria. Int Dent J. (2023) 73(2):205–11. 10.1016/j.identj.2022.03.005 35461649 PMC10023539

[16] Al-Sowygh ZH Alfadley AA Al-Saif MI SH A-W. Perceived causes of stress among Saudi dental students. King Saud Univ J Dent Sci. (2013) 4(1):7–15. 10.1016/j.ksujds.2012.11.002

[17] Sfeatcu R Balgiu B Parlatescu I. New psychometric evidences on the dental environment stress questionnaire among Romanian students. J Educ Health Promot. (2021) 10:296. 10.4103/jehp.jehp_4_21 34667796 PMC8459854

[18] Sikka N Juneja R Kumar V Bala S. Effect of dental environment stressors and coping mechanisms on perceived stress in postgraduate dental students. Int J Clin Pediatr Dent. (2021) 14(5):681–8. 10.5005/jp-journals-10005-2005 34934283 PMC8645630

[19] Cohen S Kamarck T Mermelstein R. A global measure of perceived stress. J Health Soc Behav. (1983) 24(4):385–96. 10.2307/2136404 6668417

[20] She Z Li D Zhang W Zhou N Xi J Ju K. Three versions of the perceived stress scale: psychometric evaluation in a nationally representative sample of Chinese adults during the COVID-19 pandemic. Int J Environ Res Public Health. (2021) 18(16):8312. 10.3390/ijerph18168312 34444061 PMC8391348

[21] Almadi T Cathers I Mansour H Chow AM M C. An arabic version of the perceived stress scale: translation and validation study. Int J Nurs Stud. (2012) 49(1):84–9. 10.1016/j.ijnurstu.2011.07.012 21851941

[22] Badrasawi T Nazzal Z Massad N Salameh E Ibdah A. Stress and occupational burnout levels among Palestinian dentists, and associated factors: a cross-sectional study in 2023. Heliyon. (2024) 10(11):e32034. 10.1016/j.heliyon.2024.e32034 38868015 PMC11168384

[23] Khanal S Shrestha S. Perceived stress among undergraduate students in a dental college: a descriptive cross-sectional study. J Nepal Med Assoc. (2021) 59(241):892–6. 10.31729/jnma.6446 PMC9107888 35199729

[24] Lin X-J Zhang C-Y Yang S Hsu M-L Cheng H Chen J Yu H, Stress and its association with academic performance among dental undergraduate students in fujian, China: a cross-sectional online questionnaire survey. BMC Med Educ. (2020) 20(1):e32034. 10.1186/s12909-020-02095-4 PMC7271445 32493378

[25] Juszka Z Marchewka W Smolana A Szczeklik K Olszewska-Turek K Loster B E. Loster J, Assessment of stress load among Polish and English dental students’ divisions in Poland. J Stomatol. (2021) 74(3):177–87. 10.5114/jos.2021.109185

[26] Alharbi A. Stress among dental students at qassim university. Int J Dent Sci Res. (2020) 8(1):17–21. 10.12691/ijdsr-8-1-3

[27] Meira TM Paiva SM Antelo OM Guimaraes LK Bastos SQ Tanaka OM. Perceived stress and quality of life among graduate dental faculty. J Dent Educ. (2020) 84(10):1099–107. 10.1002/jdd.12241 32535956

[28] Halboub E Alhajj MN AlKhairat AM Sahaqi AAM Quadri MFA. Perceived stress among undergraduate dental students in relation to gender, clinical training and academic performance. Acta Stomatol Croat. (2018) 52(1):37–45. 10.15644/asc52/1/6 30034003 PMC6050751

[29] Hayes A Hoover JN Karunanayake CP Uswak GS. Perceived causes of stress among a group of western Canadian dental students. BMC Res Notes. (2017) 10(1):714. 10.1186/s13104-017-2979-9 29216910 PMC5721618

[30] Mocny-Pachonska K Trzcionka A Doniec RJ Siecinski S Tanasiewicz M. The influence of gender and year of study on stress levels and coping strategies among Polish dental students. Med. (2020) 56(10):531. 10.3390/medicina56100531 PMC7600677 33053888

[31] Dasor MM Jafridin AA Azhar AA Asma AAA Manivannan PC Bilal S Yusof N Sabri BAM, Emotional intelligence, depression, stress and anxiety amongst undergraduate dental students during the COVID-19 pandemic. Int J Public Health. (2023) 68:68. 10.3389/ijph.2023.1604383 PMC9947836 36846156

[32] Amran AG Halboub E Al-Sosowa AA Al-Maweri AA SA A-M Alhajj MN. Stress dynamics in future dentists: insights from a 5-year cohort study. Eur J Dent Educ. (2026) 30(2):418–26. 10.1111/eje.13132 40401848

[33] Crego A Carrillo-Diaz M Armfield JM Romero M. Stress and academic performance in dental students: the role of coping strategies and examination-related self-efficacy. J Dent Educ. (2016) 80(2):165–72. 10.1002/j.0022-0337.2016.80.2.tb06072.x 26834134

[34] Ahmed YT Alghamdi SJ Alruwayhi SA Alsaggabi AS Alhokair AF. The effect of physical exercises on dental students’ stress. J Adv Med Dent Sci Res. (2020) 8(6):11–7.

[35] Purushothaman S Salmani D Vishnumukkala T Karikalan B Chakravarthi S Jagadeesan S Raghavan SO Gopalakrishna PK, Evaluation of sleep patterns and prevalence of stress among dental undergraduate students. J Pharm Bioallied Sci. (2025) 17(2):61–5. 10.4103/jpbs.jpbs_933_25 40859995 PMC12373367

[36] Aboalshamat K Hou XY Strodl E. The impact of a self-development coaching programme on medical and dental students’ psychological health and academic performance: a randomised controlled trial. BMC Med Educ. (2015) 15:134. 10.1186/s12909-015-0412-4 26286331 PMC4545542

[37] Alzahem AM Van der Molen HT Alaujan AH De Boer BJ. Stress management in dental students: a systematic review. Adv Med Educ Pract. 2014;5:167–76. 10.2147/AMEP.S46211 24904226 PMC4041220

