# Supplementary material for: Dental environmental stressors and their association with perceived stress among undergraduate dental students at a Palestinian University: a cross-sectional study using DES and PSS-10
Source: Front Oral Health. 2026 Sep 28;7:1952246. doi: 10.3389/froh.2026.1952246 (PMC13654717; doi:10.3389/froh.2026.1952246)
Supplement: Supplementary file 1 [file Table1.docx]

**Supplementary Material**

| **Pairwise comparison** | **Mean PSS score** | **Adjusted p-value** |
| --- | --- | --- |
| Year I vs Year II | 25.76 vs 23.58 | <0.001* |
| Year I vs Year III | 25.76 vs 25.14 | 1.000 |
| Year I vs Year IV | 25.76 vs 23.82 | 0.021* |
| Year II vs Year III | 23.58 vs 25.14 | 0.095 |
| Year II vs Year IV | 23.58 vs 23.82 | 1.000 |
| Year III vs Year IV | 25.14 vs 23.82 | 0.516 |

***Supplementary Table 1: Dunn’s post hoc pairwise comparisons for PSS score by year of study***

***Supplementary Table 2: Post hoc comparisons for DES stress categories and PSS categories***

| **Comparison** | **Chi-square** | **df** | **p-value** |
| --- | --- | --- | --- |
| Low DES vs Moderate DES | 34.99 | 2 | <0.001 |
| Low DES vs High DES | 82.71 | 2 | <0.001 |
| Moderate DES vs High DES | 38.17 | 2 | <0.001 |

***Supplementary Table 3: DES domain scores***

| **Domain** | **Valid n** | **Mean** | **SD** | **Median** | **Q1** | **Q3** |
| --- | --- | --- | --- | --- | --- | --- |
| Workload | 635 | 2.08 | 0.62 | 2.17 | 1.67 | 2.67 |
| Performance pressure | 634 | 1.89 | 0.70 | 2.00 | 1.33 | 2.33 |
| Patient treatment | 576 | 1.53 | 0.72 | 1.50 | 1.00 | 2.00 |
| Self-efficacy | 634 | 1.42 | 0.67 | 1.38 | 0.89 | 2.00 |
| Clinical training | 589 | 1.39 | 0.77 | 1.33 | 1.00 | 2.00 |
| Social stressors | 609 | 1.33 | 0.87 | 1.33 | 0.67 | 2.00 |
| Faculty and administration | 635 | 1.20 | 0.63 | 1.17 | 0.71 | 1.60 |
